# Measuring System for Synchronous Recording of Kinematic and Force Data during Handover Action of Human Dyads

**DOI:** 10.3390/s23249694

**Published:** 2023-12-08

**Authors:** Dieter F. Kutz, Lena Kopnarski, Jochen Püschel, Julian Rudisch, Claudia Voelcker-Rehage

**Affiliations:** Department of Neuromotor Behavior and Exercise, University of Münster, 48149 Münster, Germany; lena.kopnarski@uni-muenster.de (L.K.); jochen.pueschel@uni-muenster.de (J.P.); julian.rudisch@uni-muenster.de (J.R.); claudia.voelcker-rehage@uni-muenster.de (C.V.-R.)

**Keywords:** joint actions, force development, grip formation, cerebellum

## Abstract

Handover actions are joint actions between two people in which an object is handed over from a giver to a receiver. This necessitates precise coordination and synchronization of both the reach and grasp kinematics and the scaling of grip forces of the actors during the interaction. For this purpose, a measurement object is presented that records the grip forces of both actors on the instrument and allows synchronous measurement of the kinematic data of both actors and the position and orientation of the instrument in space using an optical motion capture system. Additionally, the object allows one to alter its weight in a covert fashion so that it cannot be anticipated by the actors. It is shown that the four phases of a handover, (1) reach and grasp, (2) object transport, (3) object transfer, and (4) end of handover, can be clearly identified with the described measurement system. This allows the user to measure movement kinematics and grip forces during the individual phases with high precision and therefore systematically investigate handover actions. Using exemplary data, we demonstrate in this study how movement kinematics and grip forces during a handover depend on the characteristics of the object to be measured (i.e., its size or weight).

## 1. Introduction

Skillful control of the grip forces of the fingers enables humans to perform a wide range of manipulative movements and is an essential feature of tool use in daily life [1,2,3,4]. Multi-fingered grasps offer flexibility in handling, but require a higher degree of control of individual fingers by the central nervous system than a pinch grip, as additional degrees of freedom must be controlled. The stability of a grasp can be achieved by combinations of grip forces of individual fingers [5]. Handover actions are joint actions between two persons in which an object is handed over from the giver to the receiver, which requires precise coordination of the movements and grip forces of both of them [6]. For a successful handover action, intrapersonal and interpersonal coordination in time and space are necessary [7,8]. The movement control of one partner cannot be fully predicted by the other, although we have a basic internal model of another person’s body. We can assess the current state based on visual information. Nevertheless, there remains an uncertainty of prediction for both actors, who constantly influence each other. Given this fact, it is amazing that interactions with other people in a variety of situations and with different interaction partners in daily life are extraordinarily reliable, effortless, and smooth [9]. For example, anticipating the object’s properties (e.g., its weight) can be integrated into the receiver’s plan of action for grasping the object [10]. The handover task consists of several sub-actions that use both feed-forward and feedback control mechanisms to secure a smooth object transfer, requiring predictions of motor executions and error corrections. Four phases of handover actions have been identified: (1) reach and grasp, (2) object transport, (3) object transfer, and (4) end of handover [6]. Insights into the motor control processes of both actors (giver and receiver) in handover actions, and the factors that influence them, contribute to a better understanding of human interaction and help to further develop technologies for human–robot interaction [6]. To secure a comprehensive assessment of the handover, it is necessary to measure the hand and arm movements of both participants as well as synchronously measure the grip forces of the individual participants on the handover object. A recent review [6] lists a total of nine studies in which adequate measurement systems are described. It shows that so far only two studies described measurement systems that measure both grip forces and kinematics.

For example, in 2005, Mason and MacKenzie [11] described a box with four buttons, each connected in pairs to a load cell. Each pair of buttons was the point of contact for the thumb and index finger of the giver and receiver, respectively. Kinematic data were recorded using the 3D motion analysis system OPTOTRAK (Northern Digital Inc., Waterloo, ON, Canada) with two cameras. It monitored the positions of the index fingers, thumbs, and wrists of the giver and receiver, as well as the position of the object, using infrared-emitting diodes (IREDs) wired to a control unit. This active system was certainly avant-garde in its time. Nevertheless, the necessary cables to the IREDs meant a limitation in the comfort for the participants and a limitation in the mobility of the measuring object. A further limitation resulted from the specification of the buttons as starting points for a pinch grip. This limited the grip possibilities and prevented a tri-digital prehension, which is the most frequently performed in everyday life [5,12]. A further development of this rather simple measurement object was in 2022, as described by Brand and colleagues [9]. The object could be grasped by the giver or receiver on two vertically stacked grasping surfaces of 3.5 × 2.4 cm each. This was a significant improvement over the Mason and MacKenzie measurement system, but still saw a limitation, especially for studies with elderly subjects or patients with limited finger mobility, such as patients with arthritis who could not adjust any finger aperture. The grip forces that could be applied to the grasping surface were measured using four force sensors. The object had a fixed weight of 450 g. In addition, kinematic data of the two participants as well as the movement of the object were recorded by means of a passive optical motion capture system (Vicon Motion Systems Ltd., Oxford, UK).

Handover tasks are considered to be single tasks. In everyday life, they are often performed in a dual task situation. For example, when handing over a cup of coffee, special properties of the cup are pointed out (e.g., “The cup is hot”) or a conversation is held about other content that does not relate to the object. It is amazing how well two actors perform this task without knowing the motor plan of the other person. It is therefore of interest to investigate implicit adaptations of the grasp, such as by repositioning the fingers in different task situations. It should be noted that torques can occur when holding an object upright during everyday tasks, as the load force of the object does not have to be on the vertical axis of the object and is therefore not collinear to the force of gravity. The investigation of the effect of these torques has only been considered in a few studies, e.g., [4,5,13,14]. These torques can be compensated for by increasing the grip forces of the individual fingers [4,5,13,14], which should be considered as losses. Alternatively, finger positions can be changed to improve leverage without minimizing the total grip force on an object [4,5].

The aim of this study was to develop a measurement object that synchronously measures movement kinematics and grip forces. The object should have the following additional properties:

1. It should be freely movable. Accordingly, the synchronization of the grip force measurement must be performed wirelessly.
2. The weight of the object should be able to be changed quickly and easily in the range of at least 400–1000 g.
3. The construction plan should allow for the development of measuring objects with different sizes and different grasping surfaces.

We will use example data to show that handover actions can be studied systematically in this way and to what extent, for example, properties of the object (e.g., size or weight) influence motion kinematics and grip forces. The simultaneous measurement of kinematic and force data is necessary to adequately describe the details of the actions during the handover, such as the intra-individual optimizations of the grip during each execution and their mutual influence. The measurement object presented here can be used both in a type of single task and in a dual task.

## 2. Materials and Methods

### 2.1. Reasons for the Design of the Measurement Object

A handover task consists of several subtasks and starts with the giver grasping and lifting the object [6]. The most intensively studied grasps when examining the lifting of objects by individual participants are prismatic grasps [15]. Prismatic grasps are grasps in which the thumb is opposite the fingers and the contact surfaces are parallel to each other. In such grasps, the normal forces are exerted horizontally while the load force is directed vertically and hence is manifested as the shear (tangential) force acting on the contact surface. In prismatic grasps with several fingers, the forces of the fingers opposite the thumb are reduced to a resultant force and a resultant moment of force. This is equivalent to replacing a set of fingers with a virtual finger (VF), or an imaginary finger [16,17,18]. A VF produces the same mechanical effect as a set of actual fingers. Therefore, the measurement of the grip forces can be reduced to two force–torque sensors per actuator, one force transducer for the thumb, and one for the VF.

The finger contact in a prismatic grasp can be described by the so-called ‘soft contact model’ [19]. In the soft finger contact model, contact takes place over a specific area. The fingertip deforms during contact and the point of force application can change during execution [15,20,21], which (1) increases the contact area and the magnitude of frictional torques by the fingers; (2) decreases the distances between the distal phalanx bones and the objects; and (3) allows large displacements of the force application points [15,22]. Furthermore, it is assumed that the digits do not adhere to the object and can therefore only push the object but not pull it. As a result, the digits cannot exert force couples (free moments) on the sensor in planes other than the plane of contact. An attempt to generate such a moment will result in a digit tip rolling over the contact surface. Hence, to hold the object vertically in a stationary position, the following must apply: (1) the normal force of the thumb *F^n^*th should be equal and opposite to the total *F^n^* of the opposing fingers; (2) the sum of the tangential forces *F^t^* should be equal and opposite to the gravity load *L*; (3) the *F^n^* of the thumb and opposing fingers should be sufficiently large to prevent slip; and (4) the sum of the moments exerted by all digit forces should be equal and opposite to the external moment *M* acting on the object [15]. An external moment *M* acting on the object results from holding an object during everyday tasks. In these situations, it is not always the case that the mass distribution of an object is such that the center of mass is on the vertical axis of the object and thus the load force is collinear with the force of gravity when held upright. This leads to torques that are compensated for by increasing the gripping force of individual fingers [4,5,13,14] and their positioning [4,5]. In our experiments, the positions of fingers on the surface are not predetermined but rather unrestrained and freely selectable according to the individual geometric and physiological properties of the subject’s hand. It is precisely the ability to reposition the fingers and thus change the respective lever of the finger that leads to a minimization of the forces to be applied, especially the involuntary enslaving forces [23,24,25]. This leads to changes in finger positions during the task, which can be measured using a video motion tracking system. It should be noted that during the abductions/adductions of the fingers, tangential forces are exerted on the fingertips by active torque generation at the metacarpophalangeal joints [26]. In this respect, human hands differ from today’s robotic hands. In the latter, the metacarpophalangeal joints usually consist of simple hinges and tangential loads are passively supported by the joint structures without active control [15].

### 2.2. Test Objects and Motion Tracking

**Figure 1:**
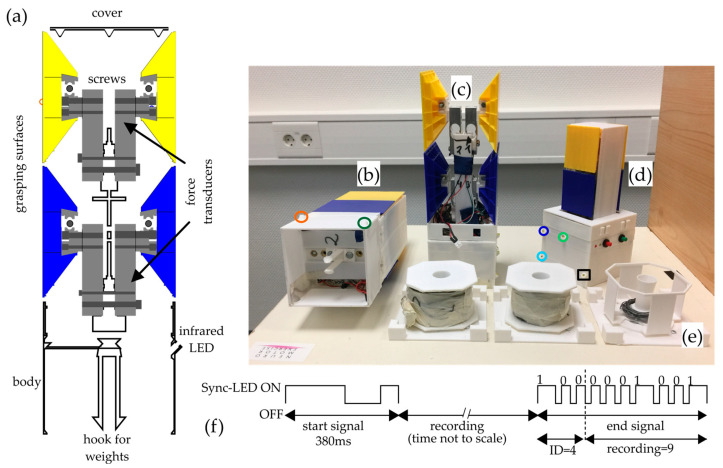
Design of the measurement objects. (**a**) Cross section of the large object. (**b**) Large object with view of the body from below and the hook system for attaching the weights. (**c**) Large object with open view of the inner structure of the upper part and the interchangeable grasping surfaces. (**d**) Small object with complete cover, as used in the experiment. (**e**) Set of exchangeable weights. (**f**) Example of the flash sequences of the Sync-LED for start signal and end signal. (**b**,**d**) Colored circles indicate the position of continuous active infrared LEDs. Colors are the same as in the graphical abstract and Figure 2. The black square in (**d**) indicates the flashing Sync-LED.

**Figure 2:**
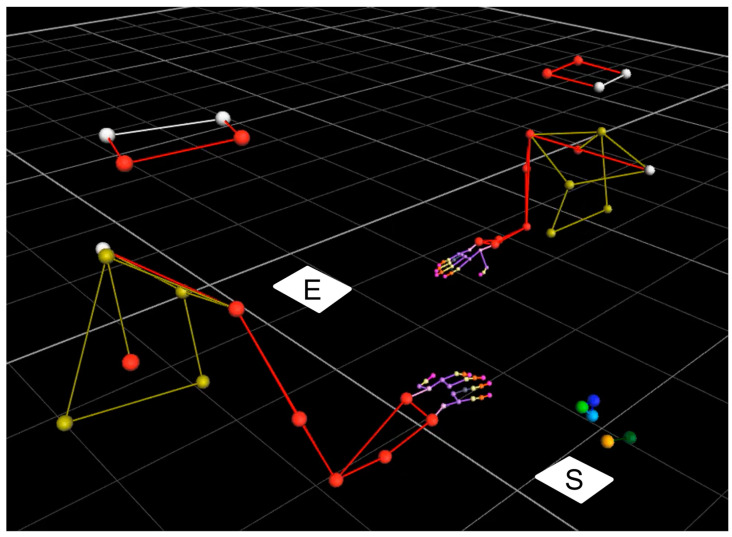
Arm–hand position of both participants (left: giver, right: receiver) at the beginning of the measurement recorded by the Vicon motion capture system. The white areas marked S and E indicate the start and end positions of the measurement object. The five markers above S indicate the position and shape of the body of the measuring object.

The test objects we designed consisted of self-constructed 3D-printed structures with control units that integrated force sensors and infrared LEDs, as well as mechanisms for weight change (Figure 1a). Two different test objects, differing in size, were developed (Figure 1b,d). The grasping surfaces, which differed in size and distance from each other (5 cm × 5 cm × 5 cm; 8 cm × 8 cm × 8 cm) between the two objects, were located above a body and were arranged one above the other. The vertical arrangement of the exchangeable mass and grasping surfaces was chosen to enable a defined grasp that could compensate for the external moment *M* due to gravity (see Reasons for the design of the measurement object) and that could be kept as constant as possible even when the transfer was repeated. The design of the individual interchangeable grasping surfaces was chosen in such a way that a high degree of rigidity was achieved against bending during grasping, and at the same time a low mass was achieved by minimizing material consumption. In addition, it was determined that the giver always uses the lower of the two grasping surfaces (blue grasping surface) and the receiver always uses the upper yellow surface (Figure 1). This results in less overlapping of the markers of the giver’s and receiver’s hands and thus a reduction in incorrect assignment of the markers. Four integrated 3D force–torque sensors (Type 1B-S, Zemic Europe B.V., Etten-Leur, The Netherlands) under the grasping surfaces allowed the grip force of the giver and receiver to be measured separately from each actor (Figure 1c). Force data could be recorded with a sampling rate of 100 Hz. Both objects had an identical body (8 cm × 8 cm × 8 cm, Figure 1b), which allowed easy and quick attachment of weights inside the object (see Supplementary Videos S1 and S2), without being able to see from the outside which weight was attached. Three different object weights (Figure 1e) were prepared, and the total weight of the whole object did not differ between small and large objects: light = 400 g, medium = 700 g, heavy = 1000 g. Six infrared LEDs were embedded in the base. Five continuous active LEDs (Figure 1b,d, positions of the LEDs are marked by colored circles) allowed the tracking of the object’s motion in a motion capture system (Vicon Motion Systems Ltd., Oxford, UK) with 10 cameras at a sampling rate of 100 Hz. Thirty-eight reflective markers were placed to measure the kinematics of the participants’ heads, torsos, arms, and hands (as an example, see Figure 2, and for references see [27,28]). To synchronize the data stream of the Vicon system with the data recording of the object, the sixth LED was a Sync-LED (Figure 1d). LED position was marked by a black square. It flashed a special pulse sequence that signaled the start and end of the recording (Figure 1f). The start signal consisted of a special sequence of 200 ms Sync-LED ON, 120 ms OFF, and 60 ms ON again. Information about the object ID and the number of the recording was also encoded in the end signal. The object ID was a 3 bit code with the most significant bit first and the following signals: 1 = 60 ms ON and 20 ms OFF, 0 = 30 ms ON and 20 ms OFF. The sequence for object ID = 4 is shown in Figure 1f. The object ID code is followed by the sequence of the recording number. The recording number was a 7 bit, with the most significant bit first and coded with the same ON/OFF duration for 1 and 0 as for the object ID. An example of the coding for recording = 9 is shown in Figure 1f (end signal, to the right of the dashed line). This form of coding was chosen to avoid misidentification of recording starts and stops, which can occur when using a permanent gating signal due to an interruption of the Sync-LED signal. Three-dimensional print files of the measurement object, the plan of the printed circuit board, and the assembly list are available in the Supplementary Materials.

### 2.3. Participants and Procedure

Forty participants (thirty-one female, nine male) aged 22.6 ± 2.5 years attended the experiment and gave written informed consent for their voluntary participation. Thirty-nine participants were classified as right-handed and one participant was classified as ambidextrous (Edinburgh Handedness Inventory [29]). A dyad consisting of two randomly selected participants was measured in one session. Reflective markers were attached to both participants’ upper bodies, arms, and hands. Participants sat opposite each other at a table with elbows bent about 90°, where the forearms were placed on the table and the palms were rested flat on the table. Participants had to take this rest position at the beginning and end of each trial. One participant was assigned the role of the giver and one was assigned the role of the receiver. After half of the trials (*n* = 60) had been conducted, roles were switched. The task of the participants was to hand over the object. The variable object size (small, large) was presented in a block design, with the order counterbalanced across all dyads. The object weight variable (light, medium, heavy) was presented in a pseudo-random order and balanced within a block. In total, each giver performed each condition 10 times (2 object sizes × 3 object weights), resulting in six blocks and 60 trials. As each receiver also played the role of the giver, a total of 2400 trials (40 participants × 60 trials) were recorded.

The participants were instructed to perform a handover action as naturally as possible. At the beginning of a trial, the object was placed on a foam pad (17 cm × 20.5 cm) fixed centrally to the table on the right-hand side of the giver (Figure 2, white square indicated with S (start position)). The test object was switched on by the experimenter by pressing the green button (Figure 1d), which started the recording and caused the Sync-LED to flash the start signal sequence (Figure 1f). After an acoustic signal, the giver grasped the object at the lower grasping surfaces (blue, Figure 1) and handed it over to the receiver, who grasped it at the upper grasping surfaces (yellow, Figure 1). The receiver then placed the object on a foam pad on the other side of the table (Figure 2, white square indicated with E (end position)), which ended the trial (see Supplementary Video S3). The recordings were ended either by pressing the green button again or automatically after 100 s. In both cases, the Sync-LED flashed the end sequence consisting of the object ID and the recording number (Figure 1f). In cases where the measurement was stopped before 100 s had elapsed, the saved data set was filled with zeros to make 10,000 measurement points.

### 2.4. Preprocessing and Data analysis

Data were preprocessed and analyzed according to the phases of a handover. For demonstration of the functionality of the measurement object, the first phase of a handover (reach and grasp, [6]) was preprocessed and analyzed. This phase started with the movement of the hand from its initial position (Position S in Figure 2) to the object (kinematic data) and ended with the contact of any finger with the object until it was lifted off (kinematic and force data). From this phase, the interval between the first contact of any finger with the object to the moment of lifting was analyzed to determine the duration of the lift delay. Differences in force development and grip formation became apparent, which affected the determination of the duration of the lift-off of the object. In the following section, two methods are described to determine the duration of the lift-off based on the force data (“finger force”) and the kinematic data (“finger distance”), respectively.

The end of the lift-off interval could be defined from the kinematic data as the moment when the object had been lifted at least 2 mm in the vertical direction. The determination of the start of the lift-off interval was more difficult. This was because individual finger movements were observable (see Supplementary Video S3) before the object had been lifted, as well as during the subsequent transport phase. An increase in the grip force could be measured immediately when any finger contacted the grasping surface. The first algorithm “finger force” detected the start of the lift-off interval as the time at which a change in grip force of more than 0.07 N was measured. The interval from this point to lift-off corresponded to the lift delay based on the force data.

For the second algorithm (“finger distance”), it must be noted that tri-digital grips using the thumb, index, and middle finger are the most common grips in everyday life [5,12,30], as long as no constraints have been imposed. Since the participants were free to choose in terms of the number of fingers forming the grip, they mostly used the thumb and both fingers (index and middle finger) to form the grip. One finger serves as the force-opposing finger to the thumb, while the other finger compensates the torque caused by the mass of the object and the position of the center of gravity in order to achieve a stable position of the object in space. The force-opposing finger is characterized by the fact that it remains spatially stable to the thumb during the transport phase, while the other finger shows higher variability in its relative position due to the required adjustments. Therefore, the “finger distance” algorithm first identifies the force-opposing finger as the finger with the lowest position variability based on the variability during the transport phase (phase 2 according to [6]) from lift-off to the moment of transfer of the object to the receiver. For the finger determined in this way, the time at which the finger–thumb distance is minimal is searched for. The search is limited to the interval from the beginning of the approach of the hand to the object until its lifting. The calculated interval corresponds to the lift delay based on the kinematic data.

For all trials, the finger counteracting the thumb force was counted. Based on the respective frequency (N_index finger_ and N_middle finger_, respectively), a simple finger index was calculated using the following:

finger index = (N_index finger_ − N_middle finger_)/(N_index finger_ + N_middle finger_) (1)

A finger index of 1.0 means that only the index finger was used to counteract the thumb force. The same applies for the middle finger at −1.0. With a value of 0.0, both fingers were used with equal frequency.

The preprocessing of the data and all statistical analyses were performed using the R 4.3.1 base package [31]. Analysis of variance was conducted using the package “ez” [32] and “apaTables” [33].

## 3. Results

To demonstrate the operation of the measurement object, we performed handover experiments with 40 dyads. To show the differences in force development and grip formation, the duration of the lift delay was determined using the two algorithms “finger force” and “finger distance”.

The results of the two algorithms separated by the factors of size (small object, large object) and weight (light, medium, heavy object) are summarized as median and inter-quartile range (IQR) in Table 1. Overall, lift delay increases with weight in both algorithms. The differences are significant for the factor of weight in both algorithms (finger force: F(1, 236) = 92.19, *p* = 0.000, η^2^ = 0.29; finger distance: F(1, 236) = 125.22, *p* = 0.000, η^2^ = 0.35). For the factor size, a difference at trend level was found for the finger force algorithm (F(1, 236) = 2.96, *p* = 0.087, η^2^ = 0.01).

Comparing the results of both algorithms, the algorithm “finger force” gives an average lift delay of 314.8 ms and the algorithm “finger distance” gives an average lift delay of 170.0 ms. This shows that force development begins even before the grip formation has reached a stable grip. In order to study the influence of the factors of size and weight, the difference between both algorithms was calculated separately for each dyad according to the size and weight of the object (Table 1, lift-delta). There is a significant difference between the start of force development and the start of grip formation for the factor weight (F(1, 236) = 22.78, *p* = 0.000, η^2^ = 0.09) as well as for the factor size (F(1, 236) = 19.73, *p* = 0.000, η^2^ = 0.08). This means that grip formation relative to force development is delayed with increasing object weight and size. It should be noted that even the smallest time difference of more than 100 ms (Table 1, lift-delta: small object/light) is too long to interpret the grip formation as a result of a reflex action at the spinal cord level.

**Figure 3:**
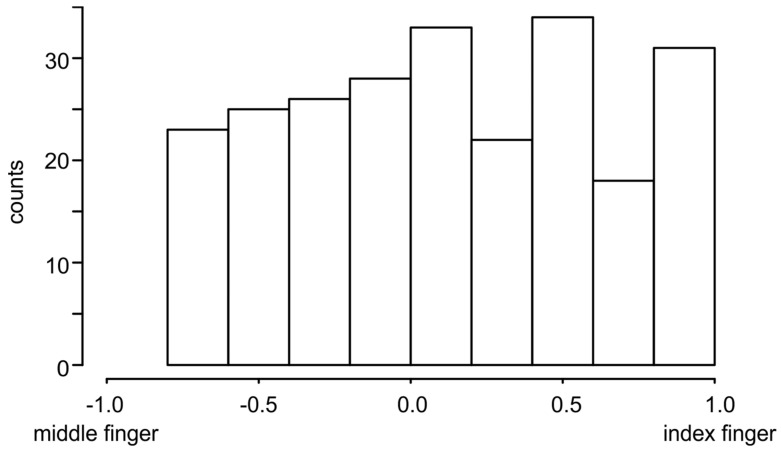
Finger index over all trials independent of size and weight of the measurement object (*n* = 240). A finger index of 1.0 means that only the index finger is used to counteract the thumb force. The same applies for the middle finger at −1.0. With a value of 0.0, both fingers are used with equal frequency.

The described differences in the duration of grip formation and force development showed that the participants adapted their grip to each combination of weight and size. To describe this individuality on a single trial basis, the finger that best opposed the force of the thumb and thus provided a stable grip was determined (see Methods, algorithm finger distance). A finger index was calculated from the frequencies of the index and middle fingers for all conditions tested. The distribution of the finger index shows that each participant performed the grip to lift the object differently each time (Figure 3). It is striking that there are no values in the range [−1.0, −0.8]. That is, for the 10 repetitions of a combination, the index finger was used at least once or twice as a force-opposing finger and no participant used only the middle finger in all repetitions. On the other hand, examination of the data revealed that 14 participants used the index finger exclusively as a force-opposing finger in at least one of the conditions. Thus, the choice of one finger as a force-opposing finger relative to the thumb is an inter-individual characteristic of the participants.

Regression analysis was performed to investigate the influence of finger choice (finger index) on the temporal difference (Table 1: lift-delta) between force development (algorithm finger force) and grip formation (algorithm finger distance). The regression analysis stratified by the factors of weight and size revealed significant differences in lift-delta in terms of dependence of finger index (Table 2). The lift-delta decreases with increasing finger index. That is, the more frequently the index finger is used as the force-opposing finger in the grip, the shorter the difference between force development and grip formation. Stratification by factor weight shows a significant increase in lift-delta with increasing object weight. Stratification by factor size also shows a significant increase at trend level (Table 2).

Altogether, the results related to lift delay show that the action of grasping an object is not a pre-programmed sequence of micro-actions whose execution is triggered by sensory stimuli. Rather, inter-individual differences in the execution and intra-individual optimization of the grip during each execution are shown to guarantee stable lifting/holding of the object.

## 4. Discussion

In this study, a measurement system has been presented that measures grip forces and kinematic data synchronously in time. The measurement object is freely movable and the weight of the object can be changed quickly and easily (Supplementary Video S2). In addition, the object can be equipped with grasping surfaces of different sizes (Figure 1). This allows the object to be adapted to different research questions (e.g., grasping objects with different surfaces) or participant collectives (e.g., old subjects, patients with limited motor skills). To demonstrate the operation of the measurement object and the necessity to assess force and motion data simultaneously, we performed handover experiments with 40 dyads (e.g., Supplementary Video S3). In order to show the differences in force development and grip formation and their effects on the data analysis, the duration of the lift delay was determined using two algorithms. Algorithm “finger force” is based on the force data (measured with the measuring object) and analyzes the force development. Algorithm “finger distance” is based on the kinematic data (synchronously measured with a motion capturing system) and analyzes the grip formation. Even though grip formation and force development during grasping are perceived as one action, the results show that force development starts at least 100 ms before reaching a stable grip (Table 1: lift-delta). We must therefore assume that they are two distinct actions controlled by different mechanisms. This can be seen, for example, in the increase in the time difference between the algorithms with the increase in weight and size (Table 1: lift-delta). Another result is that participants perform the grip differently with regard to the finger that counteracts the force of the thumb. It is remarkable that no participant used only the middle finger as a force-opposing finger during repetitions (Figure 3), while 14 participants used only the index finger. This indicates a different use of the fingers during grip formation. According to this, the index finger is more often involved together with the thumb in the formation of a stable force transmission, while the middle finger takes over other tasks to stabilize the object in space. In summary, it must be stated that the action of grasping an object is a complex interaction of the fingers used and the forces exerted over time. A singular consideration of the individual variables (force, kinematics) only insufficiently represents the action. Therefore, a synchronous measurement of these variables is necessary to comprehensively measure the nuances of the action.

The time difference between the two measures might be explained by the attempt to minimize unwanted torques. As the axis of the thumb and index finger lies above the center of gravity of the object, it is to be expected that torques will arise that have to be compensated. It has been shown that the adaptation of the grip in the first moments of grip formation is very suitable to compensate for unwanted torques [4,5,34]. In particular, young healthy subjects, as they correspond to our participants, are very fast and efficient in this adaptation performance ([5], Figure 2). In contrast, patients with cerebellar damage show significant limitations in adaptation ([34], Figure 5), which indicates the involvement of the cerebellum in the action. The cerebellum is involved in the coordination of multi-joint movements of the shoulder, arm, or finger joints [34,35,36,37]. In this process, the cerebellum integrates sensory information to form accurate predictions in self-generated movements [34,38,39]. The involvement of the cerebellum in the control of grip formation and force development would explain the temporal difference between the two actions. In addition, a simultaneous involvement of the cortex can be assumed, since both brain areas show approximately the same motor latency during finger movements [40].

The simultaneous study of force and kinematic data allows more precise insights into the complexity of grasp formation. It has been shown that the choice of finger, as a force-opposing finger in relation to the thumb, is an individual characteristic of participants. Additionally, intra-individual optimizations of grasp were evident in each execution. Further studies are needed to investigate the interaction of grip formation and force development during the subsequent phases of the handover action. Therefore, from a measurement methodology point of view, the simultaneous measurement of kinematic and force data is necessary to adequately describe the details of the actions during the handover and their mutual influence.

## Figures and Tables

**Table 1:** Median and inter-quartile ranges ^1^ of the lift delays separated by object size and weight. Note that for the two algorithms, the time specification was calculated as time before lift-off. Lift-delta was calculated based on the individual differences in both algorithms.

	Small Object			Large Object		
	Light	Medium	Heavy	Light	Medium	Heavy
finger force	226.5/65.6	275.5/74.0	359.0/119.5	244.0/60.0	303.0/98.0	373.0/180.5
finger distance	117.0/52.5	161.5/62.5	207.5/81.9	116.5/53.0	157.5/84.0	202.5/107.6
lift-delta	103.0/34.5	111.0/36.5	142.5/72.5	133.0/48.5	152.5/58.0	163.5/92.9

^1^ All values are in ms.

**Table 2:** Regression coefficients for the regression lift-delta~regression index stratified by the factors of size and weight.

Coefficients	Estimate/Std. Error, *p*
intercept	145.1/3.4, <2 × 10^−16^
finger index	−63.8/26.0, 0.01
finger index: size	23.1/12.9, 0.07
finger index: weight	18.5/7.9, 0.02
F(3, 226) = 3.755, *p* = 0.01, adjusted R^2^ = 0.03	

## Data Availability

Data are available in Supplementary File: File S5_data.txt.

## Supplementary Materials

The following supporting information can be downloaded at https://zenodo.org/records/10299104: Video: Video S1—Hook operation; Video S2—Weight change; Video S3—Handover action; file: File S4—3D-Printer_Files-PCB-Firmware for the construction of the measured object, a plan of the printed circuit board (PCB) with the assembly list, and firmware to use the controller; data file: File S5—data.txt.
